# DryMass: handling and analyzing quantitative phase microscopy images of spherical, cell-sized objects

**DOI:** 10.1186/s12859-020-03553-y

**Published:** 2020-06-03

**Authors:** Paul Müller, Gheorghe Cojoc, Jochen Guck

**Affiliations:** 1 Biotechnology Center, Center for Molecular and Cellular Bioengineering, Technische Universität Dresden, Tatzberg 47/49, Dresden, 01307 Germany; 2 Max Planck Institute for the Science of Light, Max-Planck-Zentrum für Physik und Medizin, Staudtstr. 2, Erlangen, 91058 Germany

**Keywords:** Digital holography, Quantitative phase imaging, Refractive index, Cell analysis, Cell characterization, Rytov approximation, Marker-free imaging

## Abstract

**Background:**

Quantitative phase imaging (QPI) is an established tool for the marker-free classification and quantitative characterization of biological samples. For spherical objects, such as cells in suspension, microgel beads, or liquid droplets, a single QPI image is sufficient to extract the radius and the average refractive index. This technique is invaluable, as it allows the characterization of large sample populations at high measurement rates. However, until now, no universal software existed that could perform this type of analysis. Besides the choice of imaging modality and the variety in imaging software, the main difficulty has been to automate the entire analysis pipeline from raw data to ensemble statistics.

**Results:**

We present DryMass, a powerful tool for QPI that covers all relevant steps from loading experimental data (multiple file formats supported), computing the phase data (built-in, automated hologram analysis), performing phase background corrections (offset, tilt, second order polynomial) to fitting scattering models (light projection, Rytov approximation, Mie simulations) to spherical phase objects for the extraction of dry mass, radius, and average refractive index. The major contribution of DryMass is a user-convenient, reliable, reproducible, and automated analysis pipeline for an arbitrary number of QPI datasets of arbitrary sizes.

**Conclusion:**

DryMass is a leap forward for data analysis in QPI, as it not only makes it easier to visualize raw QPI data and reproduce previous results in the field, but it also opens up QPI analysis to users without a background in programming or phase imaging.

## Background

Quantitative phase imaging (QPI) is a technique frequently used for the determination of the refractive index (RI) and related physical properties, such as protein concentration or dry mass, of biological cells and cell-sized objects [1–3]. The physical quantity that QPI measures is the optical phase retardation introduced by the sample, which depends on its three-dimensional (3D) RI distribution. Until recently, one of the main obstacles in QPI was the extraction of the RI from the measured phase. This problem has been resolved, to a large extent, by optical diffraction tomography (ODT) which takes into account multiple viewing angles to construct a 3D map of the sample’s RI. While ODT yields RI maps with subcellular resolution, it requires elaborate experimental setups and computationally expensive tomographic reconstruction algorithms [4–7]. Furthermore, for objects that are homogeneous and exhibit a regular shape, a two-dimensional (2D) phase analysis can be sufficient. For spherical protein droplets and microgel beads, this approach enables an accurate evaluation of refractive index and size [8]. For suspended cells, it can approximate the average refractive index and allows the quantification of relative differences [1, 9]. Thus, many studies favor 2D QPI as a fast and efficient way to track and quantify changes in the RI.

A prominent approach in single-cell QPI analysis exploits the fact that cells assume a spherical shape when they are put in suspension. The cell’s sphericity can be used to extract an average value of its RI from 2D phase data. Several studies have used this approach to characterize single cells, isolated cell nuclei, liquid droplets, or microgel beads [1, 3, 8–10]. While this approach is powerful and yet simple, until now a considerable amount of programming knowledge was required to perform the fundamental tasks of data analysis: loading experimental data, digital reconstruction of holograms, extraction of the phase data, identifying the objects of interest, finding a suitable strategy for background correction, and determining the average RI based on light-scattering models for spheres. To our knowledge, no open-source solution exists that can address all of these fundamental analysis tasks while at the same time providing a simple user interface.

Here, we present DryMass, a fundamental tool for QPI analysis which can perform all fundamental tasks from loading data to extracting the RI of spherical objects in an automated manner. With DryMass, it is possible to execute standard analysis pipelines for QPI analysis using an easy-to-use command-line interface.

## Implementation

DryMass is executed from a command shell. Each of the analysis steps is transparently implemented in an analysis pipeline that can be controlled via a configuration file. Several file formats are supported, such as raw in-line hologram data in the tagged image file (TIF) format or the proprietary SID4Bio TIF format (Phasics S.A., France). The digital reconstruction of holograms is done automatically and may optionally be modified by the user, with parameters such as filter type (e.g. disk, Gaussian, square), filter size, and sideband position, via the configuration file. If applicable, phase-unwrapping is performed using the approach by Herráez et al. [11] which is implemented in the scikit-image Python library [12]. An experimental data series can be loaded from a set of files in a folder or from a zip file.

**Fig. 1:**
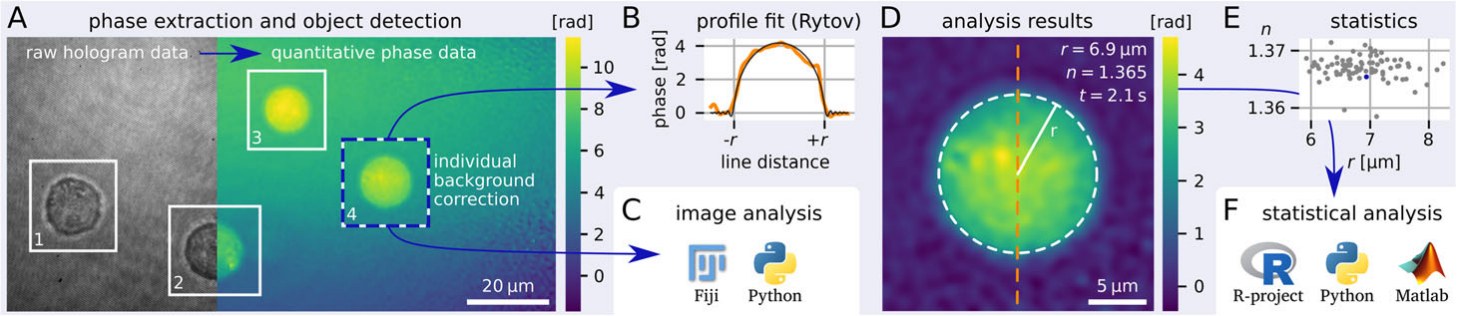
Schematic of the DryMass pipeline (shaded area) for extracting radius and refractive index (RI) of spherical objects. **a** Four HL60 cells are shown in a split-view. The left part of the image represents the raw hologram data. The right part of the image has been converted to quantitative phase data via an automated Fourier analysis. Regions of interest (ROIs) are found (white rectangles) and background-corrected for subsequent analysis steps. The cell in ROI 4 is also shown in B and D. **b** Under the assumption of sphericity, the radius *r* and the RI *n* are extracted by fitting the Rytov model (black) to the phase data (orange). (**c**) Alternatively, ROI data can be analyzed by other software. **d** Visualization of the analysis results with the Rytov approximation. The orange dashed line indicates the position of the line profile shown in (B). The white dashed circle highlights the fitted perimeter of the cell. Other parameters, such as the acquisition time *t*, are also retained. **e** For each ROI, all relevant parameters are compiled (blue data point indicates the exemplary ROI). **f** Final analysis steps can be performed with other software. The Fiji logo was kindly provided by the ImageJ community. The R-project logo is licensed CC BY-SA 4.0

Once the phase data are loaded, DryMass can automatically find phase objects, such as sparsely distributed beads or cells, and extract the corresponding ROI data (Fig. 1a). Several background correction methods are available, including the correction using a background image, a basic tilt correction [10], and a second-order polynomial correction [13]. The regions containing background data can be defined as a frame border around the ROI or via a threshold value.

Depending on the type of analysis, DryMass generates multi-page TIFs that contain phase and amplitude data for a subsequent image analysis with e.g. Fiji [14] (Fig. 1c), or a visualization of the analysis process for the user to examine. For instance, the determination of the RI for spherical objects produces a TIF that visualizes the fit via a line plot through the phase image (Fig. 1b), the corrected phase data of the original ROI (Fig. 1d), and the 2D phase residuals of the fit. The determination of the average RI for spherical cells is accomplished via the simple edge-detection method [10], the projection approximation [1], or the more accurate Rytov approximation [8]. For large data sets, DryMass produces a statistical summary, including acquisition time, RI, and object radius (Fig. 1e), which enables subsequent statistical analyses using other software (Fig. 1f). The detailed level of results makes it easy for the user to verify the analysis and, if necessary, to review the associated configuration file.

## Results and discussion

To demonstrate the working principle of DryMass, we make use of a publicly available dataset that consists of digital holograms of isolated HL60 cell nuclei measured throughout the cell cycle [15]. This dataset was first presented by Schürmann et al. [10] and shows that the RI of isolated nuclei does not vary much during the cell cycle while the increase in dry mass is counterbalanced by an increase in nuclear volume.

To analyze the dataset with DryMass, the pixel size, imaging wavelength, and medium index must be known. Normally, it is then sufficient to simply run the following command in a terminal or shell:

dm_convert data.zip

Here, *data.zip* is a file containing the raw hologram data. An interactive prompt asks the user to enter the parameters mentioned above. DryMass then creates the configuration file *drymass.cfg* in the newly generated directory *data.zip_dm* (the results directory). In addition, DryMass generates a multi-page TIF file containing the phase and amplitude data extracted from the raw data.

To extract and analyze individual phase objects, a different command is used:

dm_extract_roi data.zip

This command generates an additional multi-page TIF file containing the background-corrected ROI data. Modifications of the configuration file may be necessary and can be optimized manually in an iterative manner by re-executing the above command. In this example, we set the approximate specimen size to 7 *μ*m to reflect the small diameter of cell nuclei. For hologram analysis, we chose a *smooth disk* filter, which results in an unbiased frequency spectrum when compared to a Gaussian filter and does not exhibit ringing artifacts when compared to a plain disk filter. To detect the isolated nuclei in the extracted phase data, we employed a custom thresholding method: Since cell nuclei have a low RI and the nucleoli within have a comparatively high RI, conventional thresholding algorithms either cannot detect the nuclei reliably or segment the nucleoli only. Our thresholding method copes with this situation by disregarding the top 1% of the phase data and then taking the threshold at 20% of the resulting maximum phase relative to the mean of the original phase data. This thresholding method is implemented in DryMass and can be set via *threshold=dm-nuclei* in the *[roi]* section of the configuration. For background correction, we increased the ROI border padding to 35 pixels and employed a second order polynomial fit to correct for the skewed background phase commonly observed in digital holographic microscopy.

**Fig. 2:**
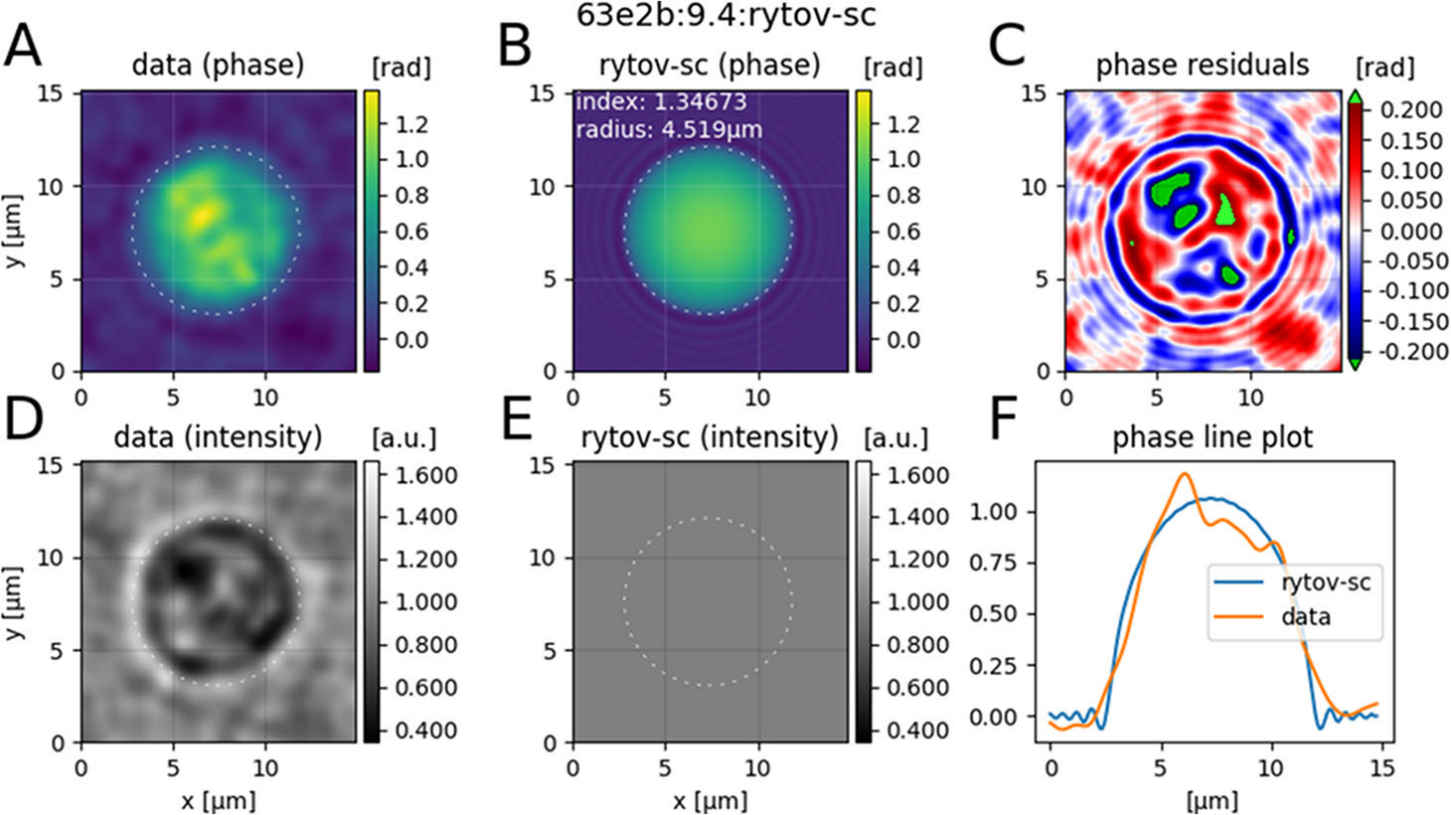
Exemplary visualization of the fitting results for a representative nucleus (stage G1, run #1) given in the stacked TIF file *sphere_image_rytov-sc_images.tif* as produced by DryMass. The title of the image contains the dataset identifier (63e2b), the image number (9), the ROI identifier (4), and the fitting method (rytov-sc: the systematically-corrected Rytov approximation [8]). The first column shows (**a**) the background-corrected phase image and (**d**) the intensity image of the isolated nucleus. The second column shows (**b**) the fitted phase and (**e**) the resulting intensity (all-one for rytov-sc). The fitted radius is shown as a dashed circle in A, B, D, and E. The last column shows (**e**) the fit residuals and (**f**) a line plot through the fitted center of the isolated nucleus for the original and the fitted phase data

Finally, to extract the RI and radius of the isolated nuclei, we chose the image fitting method using the systematically corrected Rytov approximation as described by Müller et al. [8]. To perform the sphere fit for each ROI found in the previous step, the command

dm_analyze_sphere data.zip

can be used. This command creates a multi-page TIF file containing summary plots of the fits performed. An exemplary plot is shown in Fig. 2. Please note that for each analysis step, DryMass also creates a hierarchical data format (HDF5) file, which in the case of the sphere analysis also contain the fitted phase data. The changes we made to the configuration file are available as supplement 1 (*HL60nuc.cfg*). This DryMass configuration file can be directly used to perform the entire analysis from scratch (skipping all of the commands mentioned above) using:

dm_analyze_sphere -p HL60nuc.cfg data.zip

Since some of the nuclei were not detected correctly and some of the fits failed, we manually checked each image and sorted out individual ROIs. This can easily be done by loading the TIF files produced by DryMass (see Fig. 2 for an exemplary image) in Fiji and noting down the corresponding ROI identifiers in the variable *ignore data* in the *[roi]* configuration section. The above command must be executed again for the changes to take effect. The ROIs we excluded from the analysis are given in supplement 2. A full description of the configuration file is available online in the DryMass documentation (see project home page below).

**Fig. 3:**
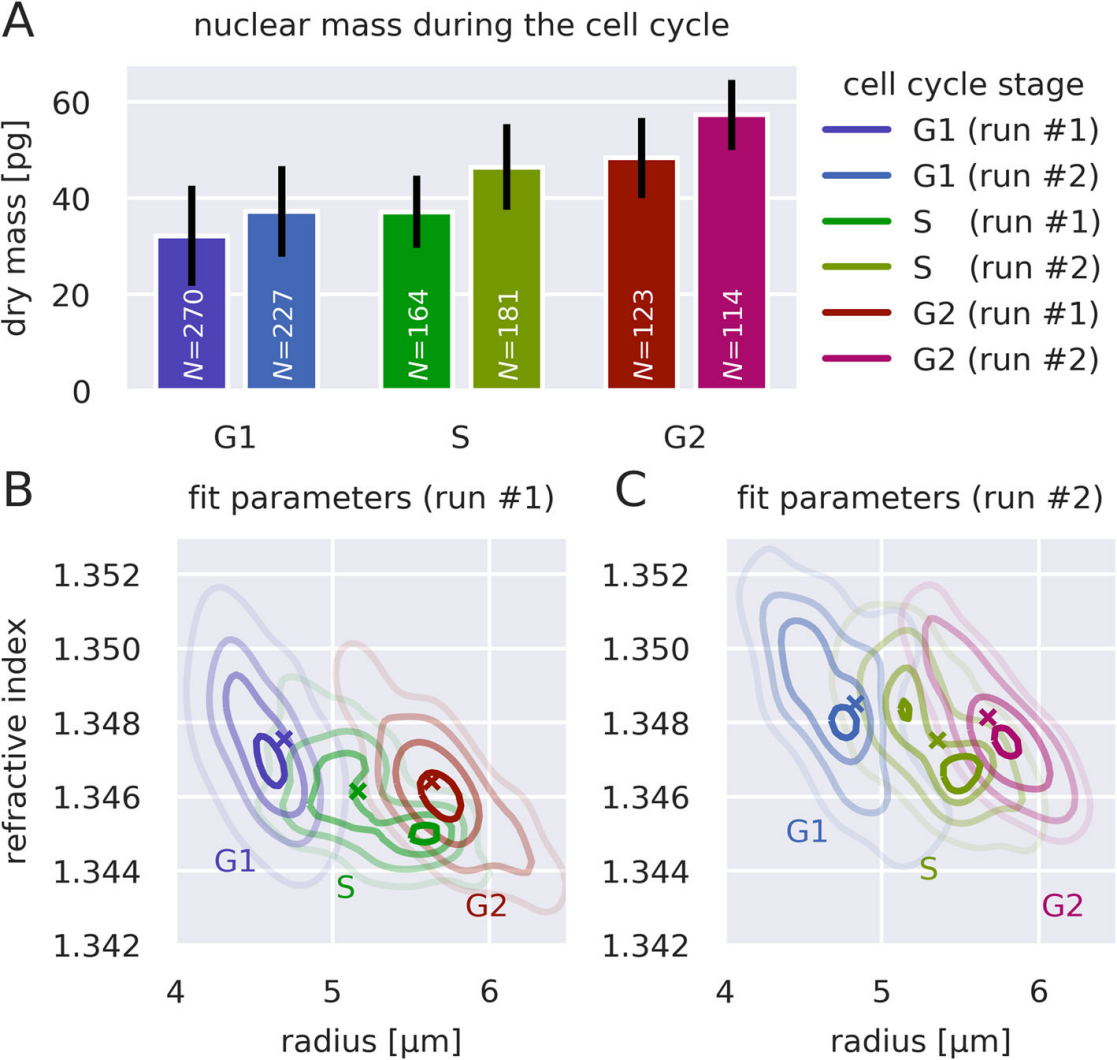
Dry mass, refractive index, and radius of isolated HL60 cell nuclei during the cell cycle. (**a**) The box plots show the mean dry mass of the nuclei throughout the cell cycle stages and for two independent measurement runs. Whiskers indicate the standard deviation. The number of isolated nuclei analyzed is displayed in white for each box. (**b,c**) The contour plots show the fit parameter distribution (phase image fit based on the Rytov approximation; see main text for details) of the two measurement runs. The color code is identical to that in (**a**). The contour lines follow a multivariate kernel density estimate and correspond to the 95th, 75th, 50th, and 25th percentiles (from dark to bright). The crosses indicate the mean value of each distribution

Overall, a total of 1079 isolated nuclei where analyzed (In the original study, 367 nuclei were analyzed manually). The results are shown in Fig. 3 and confirm the results found previously (see figure 5 in [10]). Figure 3a shows the mean dry mass of the two measurement runs for each cell cycle stage. Although the absolute nuclear dry mass varies in-between the two measurement runs, it increases as the cell progresses through the cell cycle. Figures 3b and c summarize the fitted parameters of the two measurement runs. Overall, the RI exhibits only a small change, but the radius of isolated nuclei clearly increases from stage G1 to G2. Notably, it appears as if the nuclei in stage S exhibit two populations. This observation was not made in the original analysis [10] which did not employ phase model fitting, but used the (less accurate) contour data to determine the radius of isolated nuclei (data not shown). Furthermore, the RI values presented here are considerably lower than those in the original analysis, which is a result of the more accurate analysis with the Rytov approximation as well.

The example presented above emphasizes the great potential a simple and cost-effective 2D phase analysis may have. Recent developments in RI tomography have enabled detailed 3D analyses with subcellular resolution (e.g. [16]). However, this great level of detail is not always necessary, for instance when comparing statistical distributions where only the mean values of RI, radius, or dry mass are relevant. Furthermore, a simple 2D phase analysis is not only easier to realize experimentally, but it is now also possible to automate the entire analysis pipeline with DryMass.

The present manuscript showcased only one possible use case of DryMass. The software is capable of exactly characterizing homogeneous hydrogel beads or liquid droplets. Conversely, it can be used to verify the homogeneity of artificially produced beads or to verify the optical alignment of QPI microscopes using well-known reference beads. Since DryMass also keeps track of the temporal information, it can be used for time-lapse experiments. Undoubtedly, with the capability to reuse configuration data, DryMass is well-suited for the analysis of arbitrarily large datasets.

Data analysis with DryMass is fully automated and only needs minimal supervision. Manual intervention is required when ROIs are not detected correctly. This may happen, for instance, when the analyzed objects are densely packed or when imaging artifacts occur. In such case, the user may choose to revisit the parameters for ROI detection via the configuration file or, if necessary, to manually exclude false ROIs from the analysis. In any event, the entire analysis pipeline is recorded in the configuration file and is executed in an automated manner, making it fully reproducible and less biased than methods that involve manual analysis steps.

Additional analysis methods or extensions can easily be incorporated into DryMass. For instance, important enhancements, such as the thresholding method for HL60 cell nuclei as described here, can easily be included in future releases of DryMass. Another example is the introduction of the Rytov approximation for a more accurate analysis of spherical objects [8]. For the example presented here, this novelty led to more accurate results (with the overall message remaining unchanged). The modular design of DryMass makes it straightforward to improve the software and to create new analysis pipelines based on DryMass. We welcome bug reports or feature requests and endorse external contributions via pull requests to the DryMass repository at https://github.com/RI-imaging/DryMass.

## Conclusions

DryMass is a command-line tool for the analysis and visualization of QPI data. The data analysis can be controlled via a simple configuration file and thus does not require any prior programming knowledge. The implemented QPI analysis pipelines were designed to be transparent (visualization of intermediate steps), user-friendly (sensible default paramters and comprehensive documentation), and interoperable with other analysis software. Furthermore, the modular design makes it straight-forward to support additional file formats, enhance individual analysis steps, or implement novel analysis pipelines.

## Availability and requirements

**Project name:** DryMass **Project home page:**https://drymass.readthedocs.io**Operating system:** any (no restriction) **Programming language:** Python **Other requirements:** Python ≥3.6, pip ≥19.0 **License:** MIT **Any restrictions to use by non-academics:** none

## Supplementary information

**Additional file 1** Title of data: DryMass analysis recipe for HL60 cell nuclei. Description of data: This is a DryMass configuration file, containing all information necessary to exactly reproduce the data analysis demonstrated in the present work.

**Additional file 2** Title of data: Regions of interest removed from the final analysis. Description of data: This file contains the identifiers of the regions of interest (ROI) that were not used in the data analysis for figure 3 in the manuscript. Copy and replace the respective line in the [roi] section of the corresponding DryMass configuration file (drymass.cfg).

## Data Availability

The datasets analyzed and the corresponding DryMass configuration files are available on Figshare (10.6084/m9.figshare.10257458) [15].

## Abbreviations

2D: two-dimensional
3D: three-dimensional
HDF5: hierarchical data format (library version 5)
QPI: quantitative phase imaging
RI: refractive index
ROI: region of interest
TIF: tagged image file
